# The effect of social deprivation on clinical outcomes and the use of treatments in the UK cystic fibrosis population: a longitudinal study

**DOI:** 10.1016/S2213-2600(13)70002-X

**Published:** 2013-04

**Authors:** David C Taylor-Robinson, Rosalind L Smyth, Peter J Diggle, Margaret Whitehead

**Affiliations:** a Department of Public Health and Policy, University of Liverpool, Liverpool, UK; b Institute of Infection and Global Health, University of Liverpool, Liverpool, UK; c UCL Institute of Child Health, London, UK

## Abstract

**Background:**

Poorer socioeconomic circumstances have been linked with worse outcomes in cystic fibrosis. We assessed whether a relation exists between social deprivation and individual's clinical and health-care outcomes.

**Methods:**

We did a longitudinal registry study of the UK cystic fibrosis population younger than 40 years (8055 people with 49 337 observations for weight, the most commonly collected outcome, between Jan 1, 1996, and Dec 31, 2009). We assessed data for weight, height, body-mass index, percent predicted forced expiratory volume in 1 s (%FEV_1_), risk of *Pseudomonas aeruginosa* colonisation, and the use of major cystic fibrosis treatment modalities. We used mixed effects models to assess the association between small-area deprivation and clinical and health-care outcomes, adjusting for clinically important covariates. We give continuous outcomes as mean differences, and binary outcomes as odds ratios, comparing extremes of deprivation quintile.

**Findings:**

Compared with the least deprived areas, children from the most deprived areas weighed less (standard deviation [SD] score −0·28, 95% CI −0·38 to −0·18), were shorter (–0·31, −0·40 to −0·21, and had a lower body-mass index (–0·13, −0·22 to −0·04), were more likely to have chronic *P aeruginosa* infection (odds ratio 1·89, 95% CI 1·34 to 2·66), and have a lower %FEV_1_ (–4·12 percentage points, 95% CI −5·01 to −3·19). These inequalities were apparent very early in life and did not widen thereafter. On a population level, after adjustment for disease severity, children in the most deprived quintile were more likely to receive intravenous antibiotics (odds ratio 2·52, 95% CI 1·92 to 3·17) and nutritional treatments (1·78, 1·44 to 2·20) compared with individuals in the least deprived quintile. Patients from the most disadvantaged areas were less likely to receive DNase or inhaled antibiotic treatment.

**Interpretation:**

In the UK, children with cystic fibrosis from more disadvantaged areas have worse growth and lung function compared with children from more affluent areas, but these inequalities do not widen with advancing age. Clinicians consider deprivation status, as well as disease status, when making decisions about treatments, and this might mitigate some effects of social disadvantage.

**Funding:**

Medical Research Council (UK).

## Introduction

Cystic fibrosis is the most common life-limiting inherited disease in white populations, with most patients dying prematurely from respiratory failure. Children with cystic fibrosis in the UK and in other high-income countries are usually diagnosed in the first year of their life,^1^ and subsequently need intensive support from family and health-care services.

Cystic fibrosis is of particular interest in the study of health inequalities, because it is a genetic disease and there is no social gradient in incidence of the disorder—it affects all socioeconomic groups equally (appendix). Inequalities can develop, however, in the outcomes experienced by people with the disease. People with cystic fibrosis from socioeconomically disadvantaged backgrounds, for example, die younger than do those in more advantaged social positions in the UK and the USA.^2^, ^3^, ^4^, ^5^ Between 1986 to 1994, the adjusted risk of death was 3·65 times higher in patients with cystic fibrosis in the USA with Medicaid cover (taken as an indicator of poverty) than it was in those without Medicaid cover.^2^ In England and Wales, between 1959 and 2008, Barr and Fogarty recorded an increased risk of dying later, at an age above the median of all deaths due to cystic fibrosis, in more advantaged social classes, a pattern that has persisted for more than four decades.^5^ As with other chronic diseases, this social patterning of survival in cystic fibrosis implies that social and environmental factors affect outcomes.^6^, ^7^ Inequalities in access to specialist health care might also be important, because in many health-care systems provision and use of services decreases with patients' income,^8^, ^9^ the so-called inverse care law.^10^

To gain a better understanding of when and how inequalities in outcomes develop in cystic fibrosis, we undertook a longitudinal registry study to explore the effect of deprivation on growth, nutrition, lung function, risk of *Pseudomonas aeruginosa* colonisation, and the use of major cystic fibrosis treatment modalities in a UK-wide population cohort, in the context of a universal health-care system, free at the point of use.

## Methods

### Study design and data sources

We undertook a longitudinal retrospective cohort study of individuals in the UK cystic fibrosis registry who were younger than 40 years at last follow-up, with at least one outcome measurement and a valid postcode between Jan 1, 1996, and Dec 31, 2009. The UK cystic fibrosis registry is supported and coordinated by the UK Cystic Fibrosis Trust.^11^, ^12^ The UK cystic fibrosis registry is maintained to a high standard of data quality, and is estimated to include nearly all people thought to have cystic fibrosis in the UK population^13^ and is therefore ideally suited to the study of outcomes and treatments across the whole socioeconomic spectrum in the UK (appendix).

NHS research ethics approval (Huntingdon Research Ethics Committee 07/Q0104/2) was granted for the collection of data into the UK database. The Cystic Fibrosis Trust database committee approved the use of anonymised data in this study.

### Primary outcome and covariates

The primary clinical outcomes were weight, height, body-mass index (BMI), percent predicted forced expiratory volume in 1 s (%FEV_1_), and prevalence of *P aeruginosa* colonisation. Anthropometric values were converted into standard deviation [SD] scores using the UK reference population.^14^ The primary health-care outcomes were use of treatments in the previous year (yes or no): intravenous antibiotics, supplemental nutritional support, DNase, or inhaled antibiotic treatment. Conditional on the use of intravenous treatment, we also used the log total number of days on intravenous treatment as a secondary outcome.

The primary exposure measure was a small-area-based measure of deprivation of area of residence. Postcodes were used to derive Index of Multiple Deprivation scores for the constituent UK countries^15^ and each person was allocated to a deprivation score on the basis of the first recorded postcode on entry to the dataset. Other baseline covariates in the analysis were: sex, genotype coded as the number of delta F508 alleles (0, 1, or 2), year of birth, screening status (diagnosis by neonatal screening or otherwise), and ethnic origin (white or other). Time-varying covariates were age, presence of cystic fibrosis related diabetes (CFRD), and presence of pancreatic insufficiency (ie, whether or not an individual used pancreatic enzyme supplementation). In our health-care use analyses, we adjusted for disease severity on the basis of current %FEV_1_, *P aeruginosa* status, and BMI SD score.

### Statistical analysis

Full details are provided in the supplementary appendix. Briefly, we fitted separate longitudinal models in the paediatric (<18 years) and adult (18–40 years) age ranges. We then approximated time-trends using linear functions (eg, for %FEV_1_), piecewise or broken-stick functions (weight, BMI), or quadratics (eg, any intravenous treatment), as appropriate. For instance, population weight SD score increased to about age 3 years, and then decreased subsequently (appendix). The modelling approach involved first fitting a model adjusted for age and the baseline covariates defined above, and then testing for the significance of adding deprivation. Finally, the time-varying covariates were added to the model, to assess whether the deprivation coefficient was modified. We estimated all model parameters by maximum likelihood, using linear or generalised linear mixed effects models.^16^ We used generalised likelihood ratio statistics to compare nested models, and Wald statistics to test hypotheses about model parameters. We used R (version 2.9.2) for all statistical analyses.

### Role of the funding source

The study sponsor had no role in the design, collection, analysis, or interpretation of the data, in the writing of the report, or in the decision to submit the article for publication. The corresponding author had full access to all the data in the study and had final responsibility for the decision to submit for publication.

## Results

The final dataset for weight SD scores, the most commonly collected outcome, contained information collected at 49 337 annual reviews for 8055 patients between Jan 1, 1996 and Dec 31, 2009, in the UK (table 1 and appendix). 5324 (66%) individuals had five or more follow-up measures (appendix), with a total of 48 425 person-years of follow-up. We recorded no relation between sex ratios, birth cohort, neonatal screening and deprivation status (table 1), or number of incident cases, and age at diagnosis and deprivation status (appendix). We recorded a slight trend towards fewer heterozygote delta F508 carriers (p=0·0022), more people with no delta F508 genes (p<0·0001), and a greater proportion of non-white patients with increasing level of deprivation (p<0·0001). Compared with the UK reference population, the population of patients with cystic fibrosis weighed less (SD score −0·37, 95% CI −0·43 to −0·35 [35th centile]), were shorter (–0·50, −0·53 to −0·47 [30th centile]), and had a lower BMI (–0·08, −0·11 to −0·06 [46th centile]; in models ignoring time trends).

**Table 1:** Unadjusted characteristics of study population by deprivation quintile (UK cystic fibrosis registry 1996 to 2009)

		**1 (least deprived)**	**2**	**3**	**4**	**5 (most deprived)**	**All**	**p value**
Number of patients		1537 (19%)	1563 (19%)	1591 (20%)	1736 (22%)	1628 (20%)	8055	0·0018
Observations (for weight SD score)		9500 (19%)	9706 (20%)	9708 (20%)	10 550 (21%)	9873 (20%)	49 337	<0·0001
Female sex		712 (46%)	726 (46%)	728 (46%)	825 (48%)	773 (48%)	3764 (47%)	0·38
Age in days at diagnosis (IQR)		121 (30–731)	121 (30–670)	113 (30–730)	109 (30–728)	120 (30–730)	120 (30–730)	0·39
Number of delta F508 alleles								
	2	824 (54%)	827 (53%)	822 (52%)	907 (52%)	779 (48%)	4159 (52%)	0·0022
	1	543 (35%)	556 (36%)	560 (35%)	609 (35%)	594 (37%)	2862 (36%)	0·63
	0	170 (11%)	180 (12%)	209 (13%)	220 (13%)	255 (16%)	1034 (13%)	<0·0001
Non-white		31 (2%)	31 (2%)	52 (3%)	73 (4%)	120 (7%)	307 (4%)	<0·0001
Screened		233 (15%)	272 (17%)	245 (15%)	282 (16%)	277 (17%)	1309 (16%)	0·39
Birth cohort								
	1957 to 1966	62 (4%)	49 (3%)	64 (4%)	51 (3%)	35 (2%)	261 (3%)	<0·0045
	1967 to 1976	157 (10%)	172 (11%)	182 (11%)	171 (10%)	153 (9%)	835 (10%)	0·23
	1977 to 1986	329 (21%)	384 (25%)	369 (23%)	426 (25%)	396 (24%)	1904 (24%)	0·09
	1987 to 1996	496 (32%)	478 (31%)	489 (31%)	535 (31%)	530 (33%)	2528 (31%)	0·82
	1997 to 2006	396 (26%)	393 (25%)	396 (25%)	427 (25%)	410 (25%)	2022 (25%)	0·62
	2007 to <2010	97 (6%)	87 (6%)	91 (6%)	126 (7%)	104 (6%)	505 (6%)	0·32

Data are n (%) unless otherwise stated.

Weight SD scores increased from diagnosis up to about the age of 3 years, decreasing thereafter (appendix). After adjustment for baseline factors, at diagnosis, the weight of children in the most deprived quintile was lower than that of children in the least deprived quintile (weight SD score −0·54, 95% CI −0·73 to −0·34). The deprivation gap diminished with increasing age up to age 3 years, and from then on remained constant (table 2 and appendix). A higher weight SD score was associated with male sex, screened patients, heterozygotes for delta F508, and white patients (appendix). In adults, adjusted weight-for-age was lower in more deprived groups (table 2).

**Table 2:** Summary of adjusted effects of deprivation on clinical outcomes and use of treatments in patients with cystic fibrosis in the UK

	**Patients younger than 18 years**	**Patients aged 18 years to <40 years**
Clinical outcomes*		
FEV_1_ (percentage points [95% CI])	−4·12 (−5·01 to −3·19)	−1·6 (−4·41 to 1·25)
Weight-for-age (SD score [95% CI])	−0·28 (−0·38 to −0·18)	−0·31 (−0·46 to −0·16)
Height-for-age (SD score [95% CI])	−0·31 (−0·40 to −0·21)	−0·31 (−0·43 to −0·19)
BMI-for-age (SD score [95% CI])	−0·13 (−0·22 to −0·04)	−0·12 (−0·25 to 0·01)
*Pseudomonas aeruginosa* colonisation (OR [95% CI])	1·89 (1·34 to 2·66)	1·78 (1·26 to 2·51)
**Treatments**		
Any intravenous treatment (OR [95% CI])†	2·52 (1·92 to 3·17)	1·89 (1·51 to 2·38)
Total intravenous days per year (% change [95% CI])†	15·9 (8·2 to 24)	10·6 (2·5 to 19·2)
Supplemental feeding (OR [95% CI])‡	1·78 (1·42 to 2·2)	2·38 (1·69 to 3·36)
DNase treatment (OR [95% CI])†	0·40 (0·21 to 0·72)	0·37 (0·26 to 0·52)
Use of inhaled antibiotics (OR [95% CI])†	0·66 (0·47 to 0·93)	0·40 (0·31 to 0·5)

All estimates compare the most deprived quintile to the least deprived (reference) quintile.

* The outcomes are from separate longitudinal models adjusted for time trends, sex, genotype, screening status, and ethnic origin.

† Adjusted for time trends, sex, genotype, screening status, (FEV_1_), and *Pseudomonas aeruginosa* colonisation status.

‡ Adjusted for time trends, sex, genotype, screening status, and body mass index (BMI) SD score.

The average height of individuals in the most deprived quintile compared with the least deprived quintile was also about a third of an SD score shorter in the adjusted analysis, a difference that remained constant across all ages (table 2 and appendix). In patients younger than 18 years, a bigger height SD score was statistically significantly associated with male sex and screened patients, and statistically significantly increased in white patients with age (appendix).

**Figure 1:**
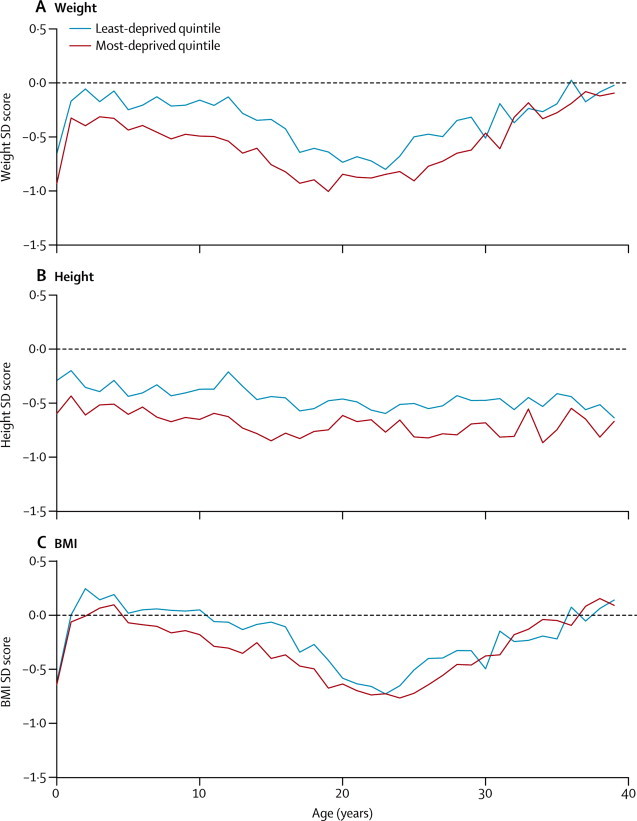
Comparison of anthropometric outcomes, by age and socioeconomic status Mean cross-sectional (A) weight, (B) height, and (C) body-mass index (BMI).

We modelled BMI SD score much like we modelled weight SD score, with a split-line at age 3 years. In the paediatric age range, there was a deprivation gap (with lower scores in the most derived groups) of −0·13 (–0·22 to −0·04; table 2). Higher BMI was associated with male sex in the paediatric age range (individuals ages 0–18 years), and had a steeper rate of decline in delta F508 homozygotes after the age of 3 years (appendix). In the adult age range, we recorded no association between BMI SD score and deprivation status (–0·12, −0·25 to 0·01; figure 1 and table 2).

Addition of the time-varying covariates did not substantially alter the deprivation effects for growth outcomes, and the estimates were consistent with a monotonic dose-response relation between deprivation and both weight and height.

**Figure 2:**
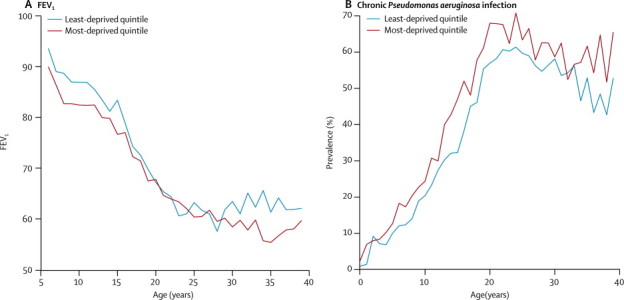
Comparison of respiratory outcomes, by age and socioeconomic status Mean cross-sectional (A) FEV_1_ and (B) *Pseudomonas aeruginosa* colonisation prevalence.

In the final model for %FEV_1_, we detected a difference of −4·1 percentage points (–5·0 to −3·1) when comparing children (<18 years) in the most deprived quintile with those in the least deprived quintile (a difference that was apparent from as soon as %FEV_1_ can be measured at about 5 years of age), but there was no evidence of an increased rate of decline in children from more deprived quintiles. Higher %FEV_1_ was associated with male sex, screened patients, heterozygote delta F508 status, white patients, no CFRD, no *P aeruginosa* colonisation, and higher BMI (figure 2 and appendix). Further adjustment for *Burkholderia cenocepacia* status and care centre did not change the deprivation effect on %FEV_1_ (appendix). The addition of BMI SD score to the model reduced the %FEV_1_ deprivation gap to −3·5 percentage points (–5·2 to −1·8). There was no statistically significant association between %FEV_1_ and social deprivation in the adult age range (table 2). The cross-sectional proportion of people with chronic *P aeruginosa* infection increased steadily with age to about 60% by the age of 20 years, and was more common in the most deprived quintile, with an odds ratio (OR) of 1·9 (95% CI 1·3 to 2·7) in the adjusted paediatric analysis for the most deprived quintile (table 2 and figure 2). An increased likelihood of *P aeruginosa* colonisation was associated with female sex, homozygote delta F508 status, CFRD, pancreatic insufficiency, and lower %FEV_1_, but adjustment for these factors did not substantially alter the deprivation effect (data not shown). The estimates were consistent with a monotonic dose-response relation between deprivation and %FEV_1_ (appendix) and risk of *P aeruginosa* colonisation (data not shown).

**Figure 3:**
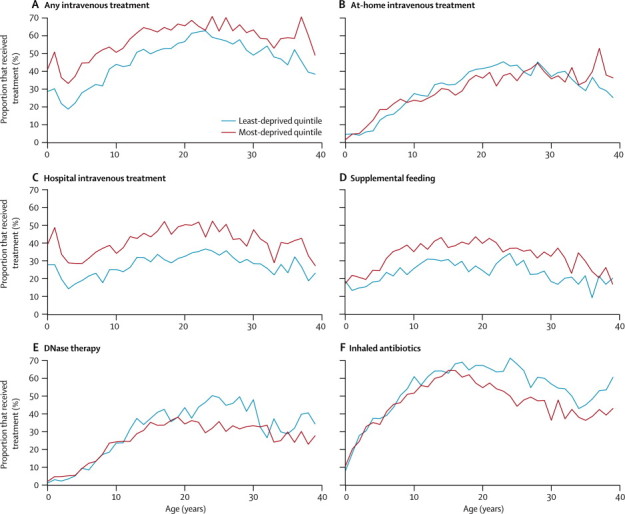
Comparison of treatment methods, by age and socioeconomic status Proportion of patients who received (A) any intravenous antibiotic treatment, (B) home intravenous antibiotic treatment, (C) hospital intravenous antibiotic treatment, (D) supplemental feeding, (E) DNase, and (F) inhaled antibiotics.

The use of any intravenous treatment, after adjustment for disease severity, was more than twice as common in the most deprived children cohort compared with the least deprived children cohort (table 2), and this deprivation difference was also present in adults (table 2 and figure 3). Further adjustment for care centre did not change this effect. Conditional on receipt of intravenous treatment, and after adjustment for disease severity, people in the most deprived quintile had more days of intravenous treatment in both the paediatric and adult age range (table 2). We analysed the receipt of hospital and at-home intravenous treatment separately and noted that the higher prevalence of any intravenous treatment seen in the most deprived quintile was almost entirely due to delivery of such treatment in hospital rather than home (figure 3). For intravenous treatment at home, the association with social deprivation was much less strong, and, in the cross-sectional analysis, at-home intravenous treatment was more common in the least deprived quintile compared with the most deprived quintile in patients between the ages of 10 years and 27 years (figure 3). Prevalence of any supplemental feeding therapy in the previous year was more common in the most deprived quintile, compared to the least, across the entire age range from age 0 years to age 40 years (OR 1·78, 95% CI 1·42 to 2·2, adjusted for baseline variables, *P aeruginosa* infection status, and BMI, in the 5–18 age group, figure 3).

We detected no statistically significant association between DNase use and deprivation in the paediatric age range before we adjusted for disease severity. After adjustment for disease severity, treatment was less likely in the most deprived quintile, in both children and adults, although the association with deprivation was stronger in adults. We saw a similar pattern for inhaled antibiotic treatment (table 2 and figure 3). The estimates were consistent with a monotonic dose-response relation between socioeconomic status and treatment outcomes (appendix).

## Discussion

Our findings show that children with cystic fibrosis from the most disadvantaged areas in the UK have lower weight, height, and BMI in the first years of life after diagnosis, are more likely to have chronic *P aeruginosa* infection, and have a lower %FEV_1_ than do children in the least disadvantaged areas. These social inequalities persist into adulthood but do not widen.

Our findings suggest evidence of positive discrimination, or so-called pro-poor bias, in the provision of some key treatments, on the basis of socioeconomic circumstances. We show that in the NHS, compared with children with cystic fibrosis in the least disadvantaged areas, children with cystic fibrosis from the most disadvantaged areas are about twice as likely, after adjustment for disease severity, to receive intravenous antibiotics (specifically in hospital) and nutritional support. Our findings also show some apparent bias in favour of wealthier populations, a so-called pro-rich bias, in two other treatments, DNase and inhaled antibiotics, with patients from the most affluent areas being more likely to receive these treatments after adjustment for disease severity.

Key strengths of this study include the population-wide coverage of the UK cystic fibrosis registry, the high quality of the data, and the longitudinal analysis. However, our study does have limitations. First, it relies on retrospective, routinely collected data and we used a standard measure of deprivation of area of residence. Each small area contains about 1500 people, and, in this respect, the Index of Multiple Deprivation scores allowed much finer resolution than US analyses^3^, ^17^, ^18^ that have used ZIP-code-linked income data, because every ZIP code contains about 30 000 people (panel).^19^ There is always the possibility of ecological fallacy (whereby inferences made at the group level do not apply to the individual), but this possibility is unlikely in view of the fact that similar associations have been seen in the US studies that use both area and individual measures of socioeconomic status.^3^, ^17^, ^18^ Second, we had valid postcodes for only 90% of the sample, although our sample size was large, with no pronounced gradient in the proportion of patients by deprivation quintile. The excluded population—those with no valid postcode—were largely older birth cohorts, owing to the improved collection of postcodes by clinical staff over time, but we do not believe that this has biased the associations detected in our analysis (appendix). Third, there is a strong cohort effect in cystic fibrosis and, with datasets of this type, age and cohort effects confound one another, and cannot be completely separated.^20^ We have adjusted for both in our analysis, to estimate the adjusted effect on deprivation.

Panel **Research in context**

**Systematic review**

We searched PubMed with the terms “(cystic fibrosis) and (inequality OR equity OR inequity OR socioeconomic OR disadvantage OR vulnerable OR poverty OR social class OR disparity)” to identify relevant studies on the effect of socioeconomic status on outcomes and treatment in people with cystic fibrosis. We applied no date or language restrictions. We identified a review that summarises all studies,^29^ much of which were done in the USA, where the health-care system is different to that in the UK. People with cystic fibrosis from socioeconomically disadvantaged backgrounds die younger than do those in more advantaged social positions in the UK^5^ and the USA.^2^ The key challenge is to understand how and when these inequalities develop, and to understand how the health-care system in the UK can mitigate or perpetuate these effects to identify promising options for intervention.

**Interpretation**

This study has identified important longitudinal differences in weight, height, body-mass index, forced expiratory volume in 1 s, and risk of *Pseudomonas aeruginosa* colonisation by deprivation in people with cystic fibrosis in the UK, which start early in life, but do not increase over time. We detected socioeconomic differences in the reported use of key treatments in the UK. People from more deprived areas are about twice as likely to receive in-hospital intravenous antibiotic treatment and nutritional support, but less likely to receive DNase and inhaled antibiotics. Interventions to reduce inequalities in outcomes in cystic fibrosis need to be focused in the antenatal period and the early years of life. Such interventions include smoking prevention and public health initiatives to address inequalities in maternal and child health. Further research is needed to clarify which elements of the cystic fibrosis care model in the UK might contribute to a reduction in the adverse outcomes associated with deprivation, and to investigate identified differences in access to inhaled treatments.

Overall, the UK cystic fibrosis population is underweight and shorter compared with the UK reference population, by about a third of an SD score. Deprivation roughly doubles this effect, lowering the SD score by another third. How much of the effect of socioeconomic status on growth outcomes is specific to cystic fibrosis, and how much is attributable to socioeconomic status effects in the general population is unclear. Comparable data in contemporary representative cohorts in the UK is absent, but the age-related changes in growth in the general population are characterised by increasing obesity in childhood from the age of 4 years onwards, with higher BMI in the more deprived populations,^21^, ^22^ findings which contrast with the patterns seen in our study. The projected weight difference at intercept in our study (–0·54) by socioeconomic status is also larger than those in other recent studies,^23^, ^24^ but direct comparison between these studies and ours is complicated by the use of different socioeconomic status measures. We speculate that having cystic fibrosis is likely to amplify the effects of socioeconomic status on nutritional status at birth and in the first few years of an individual's life.

The inequality in weight is greatest at around the time of diagnosis, and becomes narrower over the first 3 years of life. This is an important finding, because a widening of inequalities over time is often the norm.^7^, ^25^, ^26^ These findings suggest that extending the period of differential weight gain for as long as possible might reduce inequalities, further lending support to neonatal screening programmes to enable early diagnosis and treatment.^27^ We speculate that by extending this period of catch-up for as long as possible by early diagnosis (ie, screening) we might see an attenuation of the deprivation effect over time. In this study, we detected no difference in the age at diagnosis by deprivation, but screening was associated with increased weight and height and improved lung function in children. Furthermore, our finding that the prevalence of supplemental feeding treatment was higher, after adjusting for disease severity, in the most disadvantaged patients suggests that NHS professionals are actively engaged in trying to boost the nutrition of poorer patients, recognising their health disadvantage.

The socioeconomic gradient in lung function, evident as soon as it can be routinely measured at the age of 5 years, points to the crucial role of environmental and health-care factors operating in the early years of life to produce inequalities. It further reinforces the need for early diagnosis and action to prevent adverse consequences for children with cystic fibrosis living in disadvantaged circumstances. In Schechter and colleagues' cross-sectional study of US data,^2^ inequalities in %FEV_1_ by Medicaid status widened slightly from age 5 years to age 20 years. The magnitude of the inequalities in lung function at the age of 5 years seen in Schechter's study was larger (about a 9% difference) than in our UK study (4%), as was the magnitude of inequalities in lung function seen in O'Connor and colleagues' US study,^3^ which showed a difference of 5·5% between the most and least deprived quintiles. Methodological differences between the studies, however, make a direct comparison between these UK and US findings inappropriate. This study is the first to examine the relation between deprivation and %FEV_1_ in a population-level, adult cohort. We did not detect an association, despite the higher prevalence of *P aeruginosa*. We speculate that this finding might relate to the complication of progressive drop-out in older patients, and the insensitivity of %FEV_1_ as an outcome measure in adults.^20^

The increased prevalence of chronic *P aeruginosa* infection in patients from more deprived areas, after adjusting for %FEV_1_, is a new finding in a population-level cohort. In Schechter and colleagues' study,^2^ Medicaid-insured patients were more likely to have *P aeruginosa* infection than were patients who were not eligible for Medicaid insurance, but when adjusted for %FEV_1_ there was no statistically significant difference—another US cohort study did not detect an association either.^28^ Previously identified risk factors for *P aeruginosa* acquisition, which is associated with worse lung function, include female sex and genotype (both associations shown in this study), and exposure to other patients with *P aeruginosa* colonisation.^29^ Our finding that more deprived groups are more likely to receive intravenous treatment in hospital might result in more deprived patients having greater exposure to other patients with chronic *P aeruginosa*, therefore increasing their risk of infection.

We saw substantial socioeconomic differences in the reported use of key cystic fibrosis treatments in two contrasting ways. First, children from the most deprived quintile were about twice as likely to receive hospital intravenous antibiotic treatment and nutritional support, after adjustment for disease severity, compared with those from the least deprived quintile. We can speculate, from our knowledge of UK cystic fibrosis services, that clinicians in the NHS are more likely to bring children from more deprived areas into hospital for intravenous treatment because of concerns about the difficulties in delivering treatments in their homes. Conversely, children living in more affluent areas might receive intravenous treatment at home because of judgments about the adequacy of support and adherence to treatment in their home or because of their families' wish to avoid disruption to schooling and family life. This equitable model of care, with positive discrimination for socially disadvantaged children and adults with cystic fibrosis, is an uncommon finding in health systems, when access, particularly to secondary care for adults, often decreases with increasing deprivation, after adjusting for differential need.^8^, ^30^ While several studies have seen use of health services by level of deprivation to be more equal in relation to children than adults,^31^ we have detected evidence in children with cystic fibrosis that goes even further with a pro-poor bias in the NHS for specific treatments. Coupled with our findings of inequalities in outcomes by deprivation, which do not widen over time, we speculate that the treatment decisions being made by clinicians might mitigate some effects of social disadvantage. This provides encouragement that there are interventions that health services can make to reduce the adverse effects of deprivation on chronic disorders such as cystic fibrosis. In the USA, with use of ZIP-code-linked income of an area as the socioeconomic indicator, there was no gradient in intravenous treatment use in children younger than 12 years, but in young people aged 13–18 years, those living in more affluent areas were more likely to be treated (13·8% in the lowest income category compared with 19·2% in the highest).^18^

Our second, and contrasting, set of findings on cystic fibrosis treatments, however, point to an apparent pro-rich bias in two other treatments, which were more evident in adults than in children: more affluent adults in the UK were more likely to receive DNase and inhaled antibiotics than were their more disadvantaged counterparts. DNase is an expensive treatment to reduce viscosity of sputum and to aid sputum expectoration, and some evidence exists that it prevents decrease in %FEV_1_.^32^ These treatments, although expensive, are free of charge to all patients in the NHS. One possibility for the social disparity in access to them is that they are both home-based treatments, requiring regular and long-term administration. Socially disadvantaged patients with cystic fibrosis are less likely to adhere to treatments,^29^ and if they report poor adherence, clinicians might be less likely to prescribe these drugs because they are unlikely to be as effective if taken inconsistently. Evidence from the USA shows no difference in use of DNase in children by area income quintile, but Medicaid-insured children (ie, those receiving free or subsidised care) were more likely to receive DNase than were children who were not eligible for Medicaid insurance.^17^

Further research is needed to clarify which elements of the cystic fibrosis care model might contribute to a reduction in the adverse outcomes associated with deprivation. A cause for concern is the fact that the most disadvantaged families have a higher burden of treatment, in terms of time spent in hospital, which increases disruption to school and family life. Furthermore, the link with *P aeruginosa* colonisation requires further investigation. Higher socioeconomic status, as measured by parental education status, is associated with improved adherence to treatment in cystic fibrosis,^29^ and further research is needed to investigate the processes that lead to these differences. Systems to support the provision of intravenous treatment at home for more deprived groups in the UK should be explored.

Differences in access to health care cannot explain the differences in weight and height, by socioeconomic status, that are evident at the time of diagnosis, and are unlikely to explain the gradient in lung function evident at around the age of 5 years. The UK cystic fibrosis registry does not capture data about smoking in the home and these early effects might be associated with the known differences in smoking prevalence by socioeconomic status in the UK.^33^ The effect of socioeconomic status on growth in utero and in the early years of life in people with cystic fibrosis, might be mediated, at least in part, by maternal smoking, thus affecting subsequent outcomes and ultimately survival.

Future studies should focus on the assessment of interventions, such as the reduction of exposure to environmental tobacco smoke,^34^ which might mitigate the effects of deprivation during the critical early years of life, and on the identification of aspects of health-care provision in cystic fibrosis that would help overcome the extra burden of adverse consequences of cystic fibrosis faced by patients living in economically-disadvantaged circumstances.
