# Supplementary material for: The effect of social deprivation on clinical outcomes and the use of treatments in the UK cystic fibrosis population: a longitudinal study
Source: Lancet Respir Med. 2013 Apr;1(2):121–8. doi: 10.1016/S2213-2600(13)70002-X (PMC3878382; doi:10.1016/S2213-2600(13)70002-X)
Supplement: Supplementary appendix [file mmc1.pdf]

THE LANCET Respiratory Medicine

Supplementary webappendix

This webappendix formed part of the original submission and has been peer reviewed. We post it as supplied by the authors.

Supplement to: Taylor-Robinson DC, Smyth RL, Diggle PJ, Whitehead M. The effect of social deprivation on clinical outcomes and the use of treatments in the UK cystic fibrosis population: a longitudinal study. *Lancet Infect Dis* 2013; published online Jan 30. http://dx.doi.org/10.1016/S2213-2600(13)70002-X.

Online data supplement

The effect of social deprivation on clinical outcomes and the use of treatments in the UK cystic fibrosis population: A longitudinal study

David Taylor-Robinson, Rosalind Smyth, Peter Diggle, Margaret Whitehead

This data supplement contains additional information on the methods employed in this study. In addition, selected tables, plots and results are presented to aid interpretation of the results.

Methods

Design, setting and data source

The UK registry is administered by the U.K. Cystic Fibrosis Trust, and records information about the health and treatment of patients from birth. Data are now routinely collected in a standardized fashion at over 50 British cystic fibrosis specialist centres. Patients attending the British centres are seen in the outpatient clinic for a comprehensive annual review, including evaluation of clinical status, pulmonary function, microbiology of lower respiratory tract secretions, and use of CF major CF related therapies.

The registry data have been used previously in a number of epidemiological studies in CF[1, 2]. The data "cut" utilised in this study contains data collected between 1996 and 2010, and has been through rigorous quality control by data managers at the CFTrust, and external consultants at Imperial College, London, who prepare the annual review reports. This includes screening for removal of duplicates, and tracking of patent transition from paediatric to adult centres. Deaths are verified by checking with ONS. In 2000, the dataset was estimated to contain biographical information on over 92% of the estimated UK CF population[3], and registrations have increased year on year subsequently. Furthermore the CFTrust have written to every paediatrician and adult chest physician in the UK to obtain data on CF patients, and on this basis the estimated coverage is above 99% (personal communication, Diana Bilton).

The UK registry started as the UK CF Database, which was established at the University of Dundee, Scotland in 1995. Initially data were collected from 56 paediatric and adult CF clinics, using standardised forms, and validated through the system of double data entry, range checking and error correction[3]. In 2005 the data collection system changed from a paper based return system to utilise the online PortCF software used in the US registry. During this transfer there was extensive retrospective data cleaning and checking, undertaken by independent contractors. The UK CF Registry and its current software programme, Port CF, is now in its fifth year with the production of four annual reports[4]. Data are collected in over 200 fields, and the number of patients for whom a complete data set was recorded was 82% in 2009, and this has increased year on year[5]. The coverage for core variables such as weight and %FEV1, used in this analysis is higher, and almost all of the people fulfilling the study inclusion criteria had data in these fields (figure E1).

Entry criteria

The analysis did not include data for people aged >=40 years for pragmatic reasons. Only a small proportion of data are available for people over the age of 40: 5% of the annual reviews occurred in patients >=40 years. Including this data would extend the age range for the analysis up to 78 years of age. We chose to apply an upper age limit to the analysis since we have previously shown that random intercept and slope models make unrealistic assumptions when applied over long periods[6].

Primary outcome and covariates

Pulmonary function tests were performed according to international recommendations[7], measuring forced expiratory volume in one second, expressed as a percentage of predicted values for sex and height using reference equations from Wang or Hankinson[8, 9]. Supplemental nutritional support included patients receiving supplements orally, by nasogastric tube, gastrostomy tube, jejunal tube or total parenteral nutrition (TPN). Any inhaled antibiotic therapy included Tobramycin solution for inhalation, other inhaled aminoglycoside, Colistin and Promixin.

Deprivation scores as a measure of small area SES

The indices of multiple deprivation in the UK are widely used as measures of SES in epidemiological studies[10-12] and are recommended for tracking health inequalities in UK government statistics[13]. Indices of multiple deprivation combine economic, social and housing indicators measured at the census into a

composite deprivation score for small areas in the UK constituent countries[14]. There were 41500 of these small areas in the UK, containing on average 1400 people (range 500-3700). All of these small areas were ranked on the basis of the continuous deprivation score, and then divided into fifths, or 'quintiles', providing the following approximate cut-off points for normative deprivation quintiles: <8.31; 8.32 to 13.81; 13.82 to 21.20; 21.21 to 34.11, >34.11. The IMD methodology allows much finer resolution than analyses using ZIP codes in the USA, which contain on average 30 000 people[15].
We used the postcode first recorded at entry to the dataset to link an individual to an IMD score, in order to generate a fixed measure of SES.

Statistical Methods

Statistical analysis was undertaken using R (version 2.9.2 for mac), and the lme4, survival, Hmisc, memisc, mcgv and ggplot2 packages.

Kaplan-Meier estimates and Cox regression were used to assess the effect of deprivation on time to diagnosis. For the analysis of continuous outcomes (e.g. weight, height, BMI, %FEV1, IV days), we first visualised the data using spaghetti plots of individuals' measurement sequences together with non-parametric smoothed means, in order to determine the provisional model mean trajectory (see figure E10). In order establish the shape for time trends we plotted the unadjusted population average trend, and looked at the GAMs. We then approximated these trends using linear functions (e.g. %FEV1 in the younger age group), piecewise or broken-stick functions (weight, BMI), or quadratics (e.g. any IV therapy) as appropriate. This is illustrated in figure E5 below.

Repeated measures on individuals are correlated, and this must be accommodated to obtain valid inferences. To analyse the continuous-valued outcomes (weight, height and FEV_1) we used a linear mixed model[16]. Specifically, denoting by Y_{ij} the jth repeated measurement on the ith individual and t_{ij} the age at the time of measurement, we assumed that $Y_{ij} = \mu_{ij} + U_i + V_i t_{ij} + Z_{ij}$, where the μ_{ij} are the expectations of the Y_{ij} and are described by a multiple linear regression model, the (U_i,V_i) pairs are subject-specific intercepts and slopes, modelled as zero-mean bivariate Normally distributed random variables independently realised for different subjects, with means zero, variances σ_u^2 and σ_v^2 and correlation ρ, and the Z_{ij} are residuals modelled as mutually independent, Normally distributed random variables with mean zero and variance τ^2. This special case of the linear mixed model implies that the variance of the Y_{ij} increases with age, t, as the quadratic function $\tau^2 + \sigma_u^2 + \sigma_v^2 t^2$.

To analyse the binary outcomes (PA status, use of therapies in past year), we used a generalized linear mixed model. This specifies a logistic regression model for the effects of covariates on the probability of, for example, pseudomonas acquisition, but adjusts the standard errors of the regression parameters to take account of the correlation structure of the repeated measurements in the same way as described above for the linear mixed model.

We first examined univariate associations between covariates and the population mean outcomes over time, then developed a multivariate model and assessed the need for interactions. We also explored treating deprivation as a continuous term or as a factor. Although IMD is measured on a continuous scale, for descriptive summaries we have followed the common practice of grouping IMD into quintiles. However, reducing IMD to a categorical variable loses information, and also leads to models that are difficult to interpret, especially when this five-level categorical variable interacts with non-linear time effects. Where we retained IMD as a continuous variable, the fitted beta coefficients for IMD score were then used to summarise the effect of deprivation by comparing a person in the mid-point of the most deprived quintile to one in the mid-point of the least deprived quintile.

Results

Population characteristics

Figure E1: Flowchart showing people included in the primary weight analysis. After applying eligibility criteria there was very little missing data in the final complete case analysis. An age based cut off is used to stratify the analysis, and people with data straddling 18 years of age can thus contribute to both analyses.

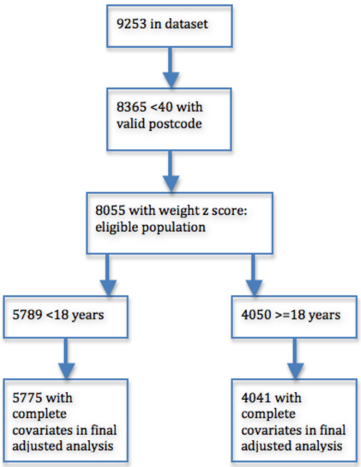

Table E1. Comparison of characteristics of eligible population versus those not meeting the inclusion criteria aged<40.

	Excluded	*Included*
Total (%)	1198	8055
Female (%)	587 (49)	3764 (46.7)
Male (%)	611 (51)	4291 (53.3)
No. delta 508: 2 (%)	621 (51.8)	4159 (51.6)
No. delta 508: 1 (%)	356 (29.7)	2862 (35.5)
No. delta 508: 0 (%)	221 (18.4)	1034 (12.8)
Birth cohort >1957-01-01 (%)	77 (6.4)	261 (3.2)
>1967-01-01 (%)	235 (19.6)	835 (10.4)
>1977-01-01 (%)	415 (34.6)	1904 (23.6)
>1987-01-01 (%)	300 (25)	2528 (31.4)
>1997-01-01 (%)	143 (11.9)	2022 (25.1)
>2007-01-01 (%)	28 (2.3)	505 (6.3)
White (%)	1155 (96.4)	7748 (96.2)
%FEV1 at age 6 (median and IQR)	94.29 (80.26 - 103.70)	92.93 (80.26 - 103.70)
% with pseudomonas at age 6 (95% CI)	8.8 (4.5 – 16.5)	10.9 (9.5 – 12.6)

Figure E2. Data follow-up in the population aged <40 years. 66% of people had 5 or more follow up measures, with a mean number of follow-up measures of 6.1 (SD 3.3).The number of annual reviews increases up to year 2000, and stabilises subsequently.

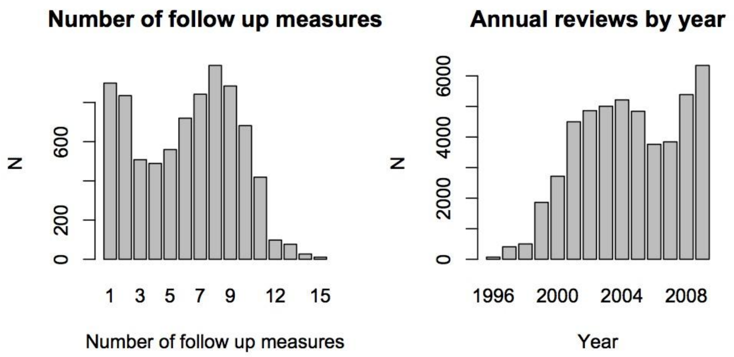

Figure E3: Distribution of incident cases (n=2066) by deprivation quintile. Error bars represent 95% binomial confidence intervals

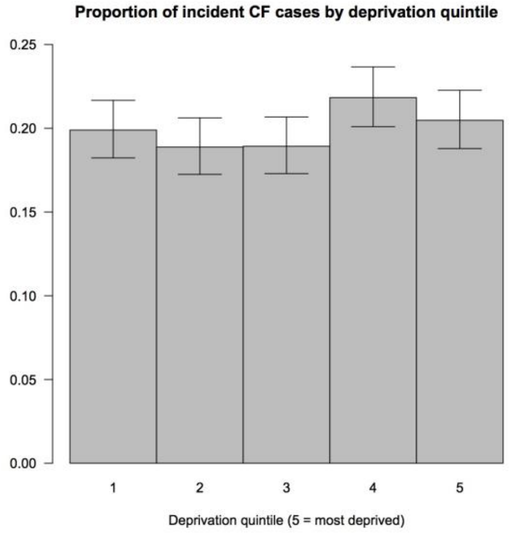

Figure E4: KM plot of time to diagnosis by deprivation quintile. This includes screening and symptomatic diagnoses.

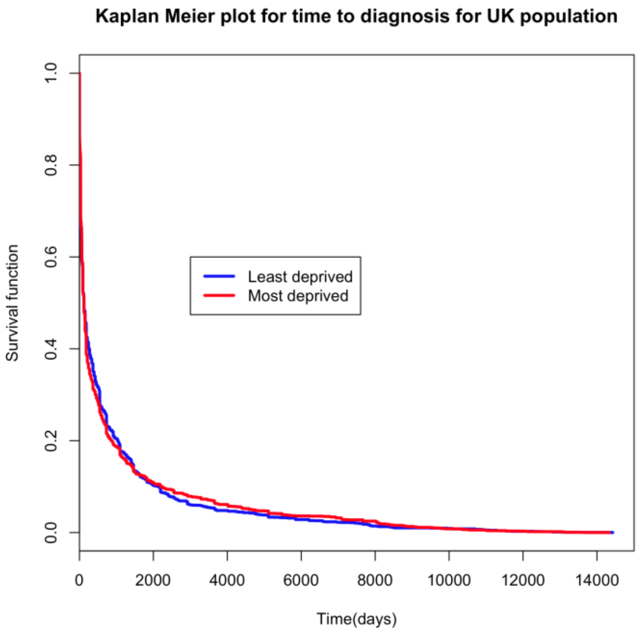

Table E2. Final linear mixed effects regression models for growth in the <18 age group. The deprivation effect is multiplied by 58 to generate the contrast between the mid point of the least and most deprived quintiles

	weight_young	*height_young*	*bmi_young*
Constant	-0.76679*** (0.09124)	-0.69634*** (0.07615)	-1.43559* (0.59086)
age	0.17231*** (0.01769)	0.01154*** (0.00296)	0.15798*** (0.02777)
age2	-0.18983*** (0.01838)		-0.17570*** (0.02880)
Number of F508 alleles: 0/2	0.06228 (0.10612)	-0.05825 (0.07068)	-0.03069 (0.15020)
Number of F508 alleles: 1/2	0.01447 (0.06184)	-0.00004 (0.04431)	-0.21288* (0.08857)
Male	0.03527 (0.05724)	0.17969*** (0.04094)	-0.09166 (0.08159)
Non-white	0.33507* (0.14012)	0.23514* (0.10062)	-0.02379 (0.18750)
Screened	0.28633*** (0.06427)	0.27879*** (0.05056)	0.10080 (0.08734)
Deprivation score	-0.00932*** (0.00169)	-0.00526*** (0.00086)	-0.00229** (0.00079)
Age x Number of F508 alleles: 0/2	-0.04279 (0.03088)	0.00184 (0.00529)	0.00141 (0.05455)
Age x Number of F508 alleles: 1/2	-0.02331 (0.01687)	0.00582 (0.00347)	0.05660 (0.03192)
Age x male	0.05831*** (0.01568)	-0.01218*** (0.00331)	0.11384*** (0.02947)
Age x non-white	-0.17607*** (0.04040)	-0.02756*** (0.00804)	-0.12099 (0.07002)
Age x screened	-0.06239*** (0.01666)	-0.01710*** (0.00407)	-0.03067 (0.03215)
Age x Deprivation score	0.00168*** (0.00045)		
age2 x Number of F508 alleles: 0/2	0.04497 (0.03199)		0.00036 (0.05688)
age2 x Number of F508 alleles: 1/2	0.03611* (0.01755)		-0.04850 (0.03332)
age2 x male	-0.07335*** (0.01636)		-0.13381*** (0.03079)

age2 x non-white	0.16692***		0.13337
	(0.04254)		(0.07400)
age2 x screened	0.06005***		0.03464
	(0.01769)		(0.03407)
Age2 x Deprivation score	-0.00173***		
	(0.00047)		
Log-likelihood	-25528.81159	-22657.81459	-28158.12899
Deviance	51057.62317	45315.62917	56316.25798
AIC	51167.62317	45409.62917	56428.25798
BIC	51623.19488	45798.52902	56891.62221
N	29235	28983	28980
Groups	5775	5750	5745

$^{*}p < 0.05$, $^{**}p < 0.01$, $^{***}p < 0.001$
Standard errors in parentheses, birthyear coefficients not shown
The deprivation effect is multiplied by 58 to generate the contrast between the mid point of the least and most deprived quintiles
age2 is the coefficient for the split line at age three in the weight and BMI analysis

Figure E5: Piecewise modelling approach to weight z score trajectory fitted by OLS compared to smoothed mean. The smoothed mean weight z score increases to around age three and decreases subsequently. This was modelled as a piecewise regression, with a 'knot' at age three. A similar approach was taken to modelling BMI z score.

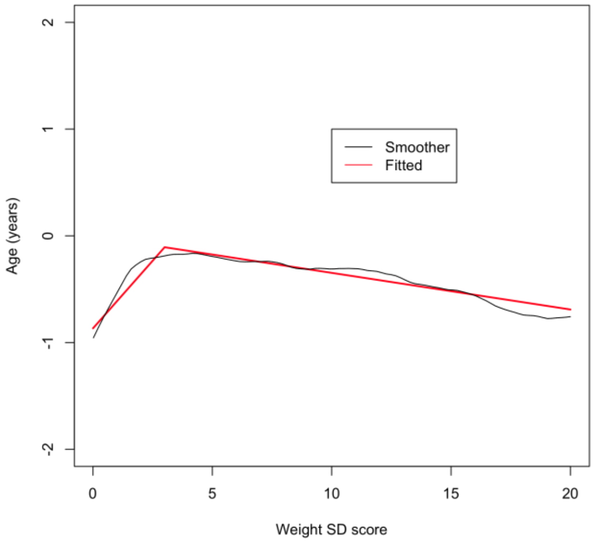

Figure E6: Modelled growth trajectories for children, comparing least (blue) and most deprived quintiles (red). These plots illustrate the contrast between deprivation quintiles. The trajectories are plotted at the reference values for other covariates in the final regression models: female sex, homozygote delta F508 carrier, not diagnosed by screening, white, born in 1991. Weight SD scores increased from the time of diagnosis to around age three, and then decreased. This is modelled as a split straight line with a knot at age three.

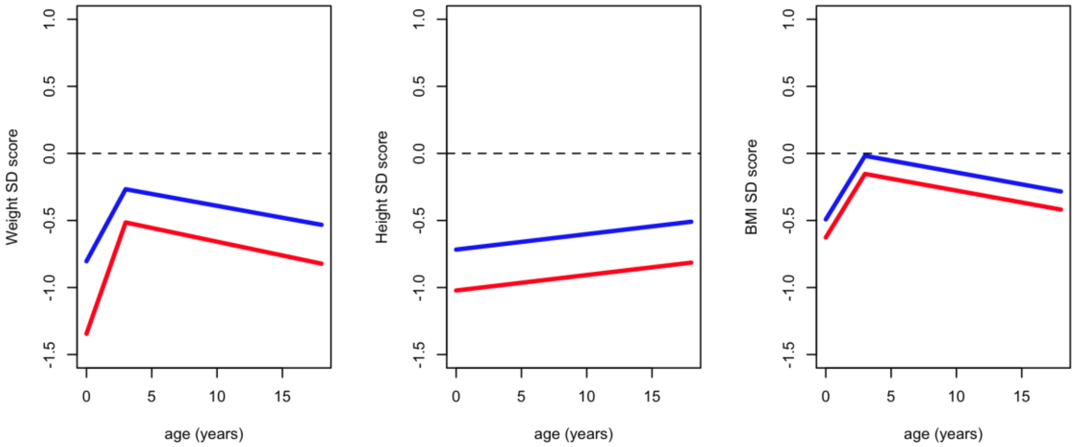

Figure E7: Weight for age versus age, illustrating the effect of sex, screening status, delta F508 carrier status, and ethnicity. Trajectories plotted at reference values for other covariates in the final regression model as above.

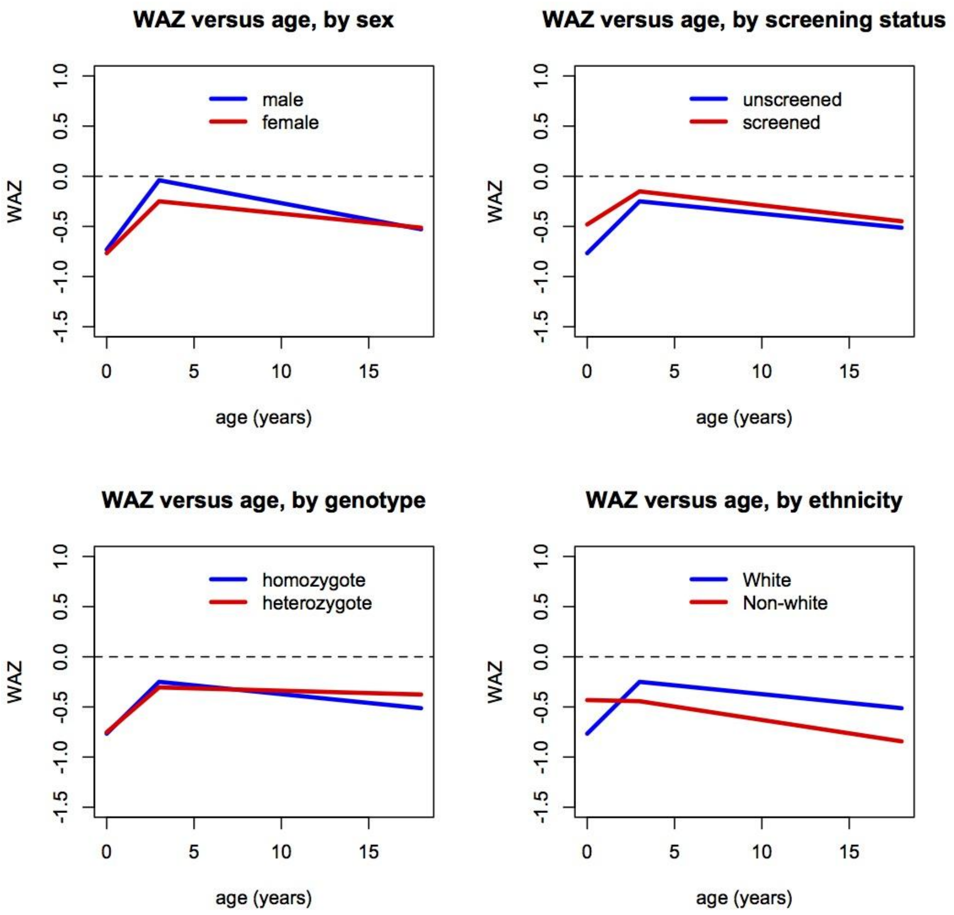

Figure E8: Height for age versus age, illustrating covariate contrasts from the final longitudinal models. Trajectories plotted at reference values for other covariates in the regression model as above.

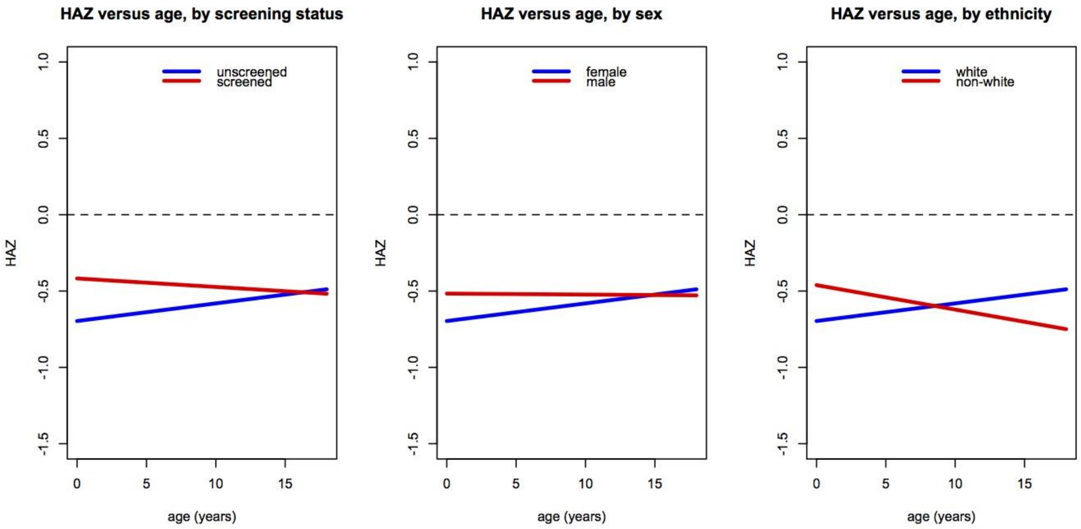

Figure E9: BMI for age versus age, illustrating covariate contrasts from the final longitudinal models. Trajectories plotted at reference values for other covariates in the regression model as above.

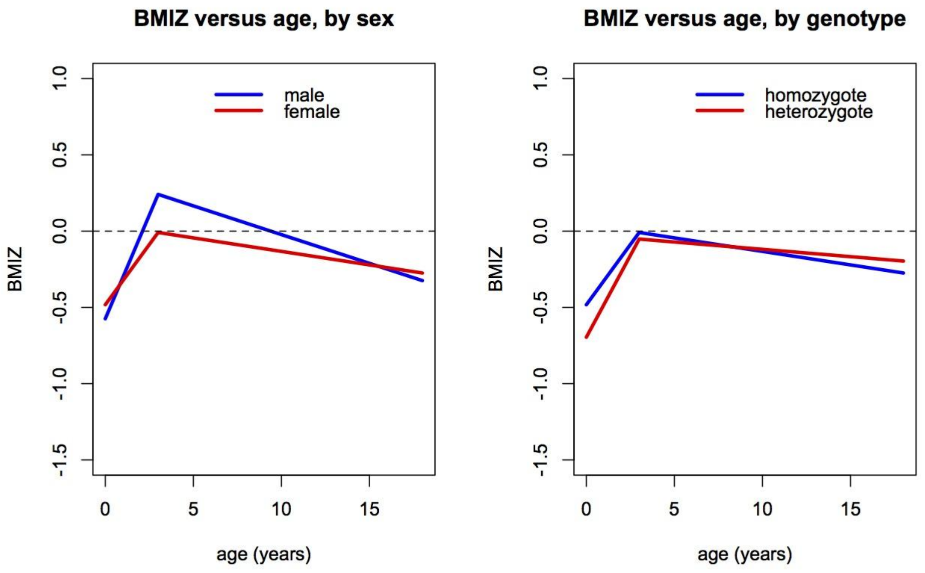

Figure E10. Spaghetti plot for %FEV1 versus age illustrating the longitudinal nature of the data. The smoothed population average is shown in the red, and randomly selected individual trajectories are in black.

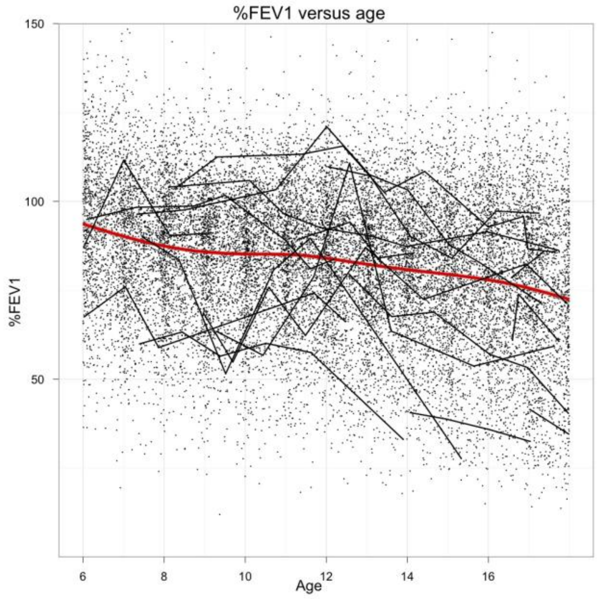

Table E3. Final regression models for %FEV1 in <18 age group.

	final	*Add time varying*	*Add BMI*
Constant	95.688***	95.568***	95.794***
	(1.276)	(1.557)	(1.529)
Age-5	-1.719***	-1.539***	-1.541***
	(0.085)	(0.136)	(0.133)
Number of F508 alleles: 0/2	-0.142	-0.258	-0.980
	(1.402)	(1.402)	(1.411)
Number of F508 alleles: 1/2	-0.258	-0.346	-0.062
	(0.829)	(0.828)	(0.835)
Male	-0.924	-0.909	-1.355
	(0.768)	(0.764)	(0.770)
nonwhite	-7.256***	-7.114***	-8.680***
	(2.149)	(2.134)	(2.147)
Screened	-3.599***	-3.584***	-3.435**
	(1.064)	(1.057)	(1.065)
Age-5 x nallele: 0/2	0.016	-0.003	0.135
	(0.161)	(0.161)	(0.157)
Age-5x nallele: 1/2	0.242*	0.233*	0.180
	(0.100)	(0.100)	(0.097)
Age-5 x sex: Male/Female	0.293**	0.274**	0.352***
	(0.094)	(0.093)	(0.090)
Age-5 x nonwhite	0.353	0.361	0.742**
	(0.266)	(0.265)	(0.257)
Age-5 x screened	0.634***	0.621***	0.585***
	(0.131)	(0.130)	(0.126)
Deprivation score	-0.071***	-0.070***	-0.061***
	(0.016)	(0.016)	(0.015)
Pseudomonas colonisation		-2.282***	-2.548***
		(0.609)	(0.602)
CFRD		-5.373**	-7.269***
		(1.980)	(1.945)
Pancreatic insufficiency		0.380	-0.094
		(0.954)	(0.945)
Age-5 x Pseudomonas colonisation		0.056	0.115
		(0.073)	(0.072)
Age-5 x CFRD		0.212	0.396*
		(0.198)	(0.194)
Age-5 x Pancreatic insufficiency		-0.129	-0.024
		(0.114)	(0.112)
BMI Z score			-2.784***
			(0.107)

Age-5 x BMI Z score			0.806***
			(0.027)
Log-likelihood	-80509.181	-80458.674	-79894.252
Deviance	161018.362	160917.348	159788.503
AIC	161100.362	161011.348	159886.503
BIC	161424.362	161382.763	160273.668
N	19979	19979	19957
Groups	4445	4445	4443

$^{*}p < 0.05$, $^{**}p < 0.01$, $^{***}p < 0.001$
Standard errors in parentheses, birthyear coefficients not shown
The deprivation effect is multiplied by 58 to generate the contrast between the mid point of the least and most deprived quintiles
age2 is the coefficient for the split line at age three in the weight and BMI analysis

In a supplementary analysis we tested for an SES and screening interaction, and although the point estimate was in the direction that supports a narrowing of inequality with screening, it was not significant.

Figure E11: Generalised additive models (GAMs) showing the shape of the relationship between %FEV1 (upper panel), risk of any IV therapy (lower panel), and deprivation score. %FEV1 decreases with increasing deprivation, and there is a dose-response relationship. Risk of any IV therapy increases with increasing deprivation, also in a graded fashion.

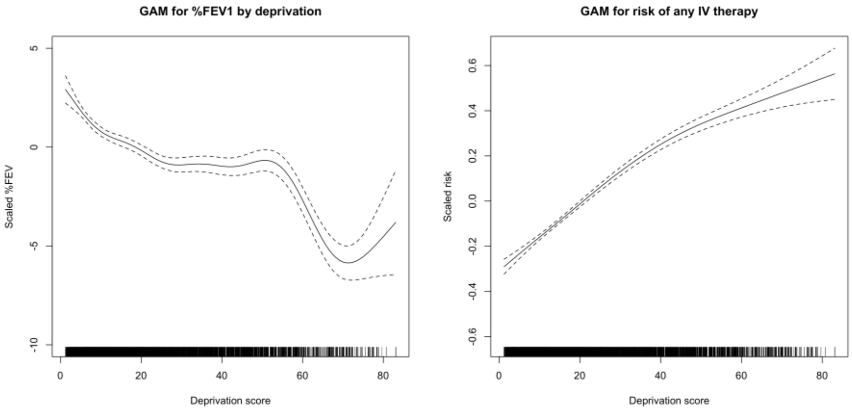

Table E4: Regression coefficients from exploratory models for %FEV1 and any IV therapy fitting deprivation as a five level factor

	Estimate	*SE*
%FEV1		
quintile 2	-0.90	0.85
quintile 3	-1.56	0.86
quintile 4	-1.33	0.83
quintile 5 (most deprived)	-3.40	0.84
Log-odds any IV therapy		
quintile 2	0.08	0.12
quintile 3	0.19	0.12
quintile 4	0.43	0.11
quintile 5 (most deprived)	0.80	0.12

(least deprived quintile 1 is the reference category)

Figure E12: Modelled trajectories for %FEV1, comparing least (blue) and most deprived quintiles (red). Trajectories plotted at reference values for other covariates in the final regression model

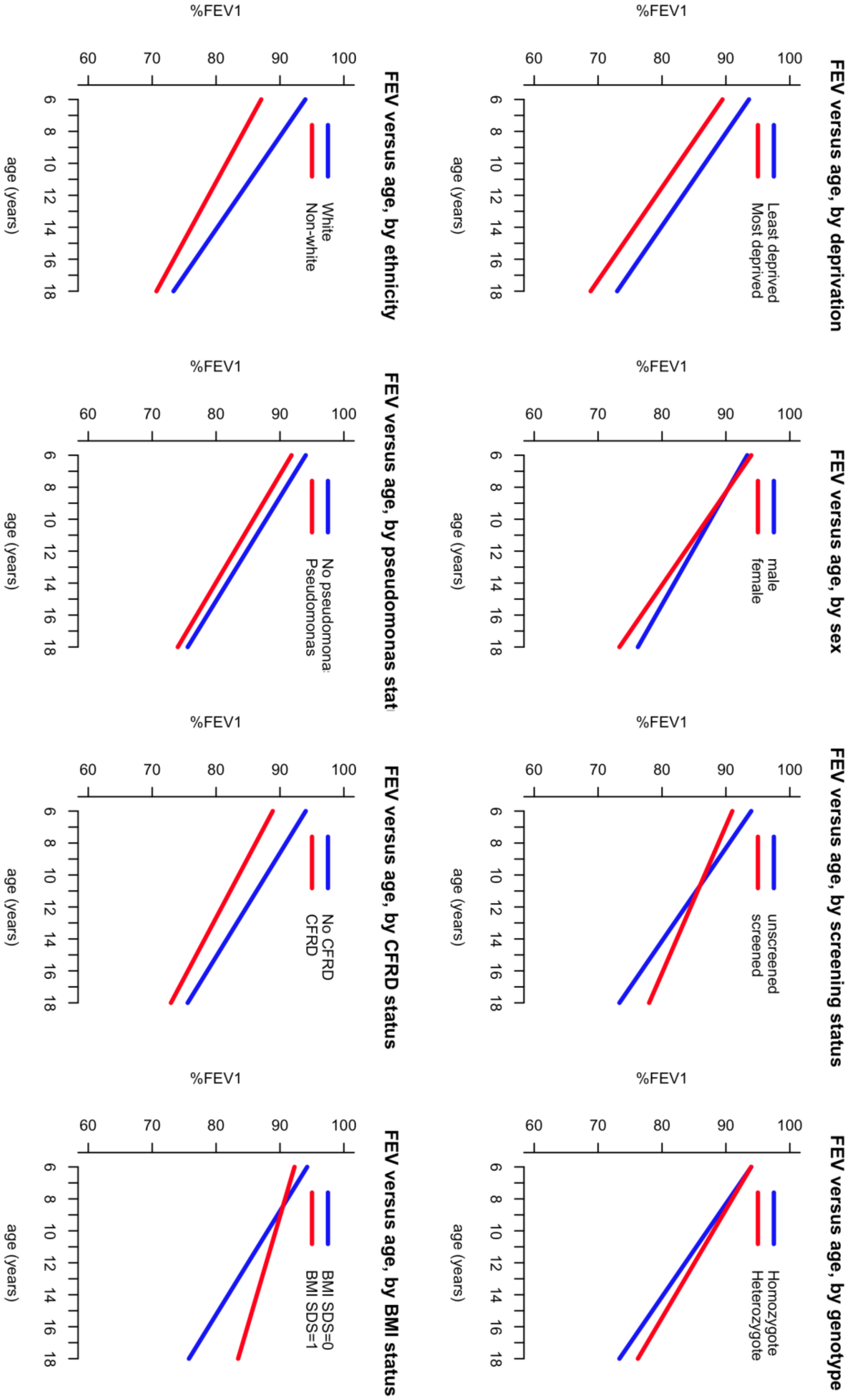

Robustness checks

Changing deprivation scores

Over the study period 18% of eligible individuals had more than one postcode recorded. As a robustness check, we repeated the analysis for %FEV1, treating SES as a time-varying covariate, but this did not materially alter the result (table E5).

Adjustment for clustering by CF centre

Differences between centres may mediate some of the effects of socioeconomic status on outcomes, and explain some of the differences in treatments received. In order to explore this we replicated the final models for %FEV1, and for any IV therapy, adding in care centre as a fixed effect. This made no difference to the deprivation effect (table E5).

Excluding data pre-2000

Excluding the data pre-2000, when recruitment to the cohort was increasing over time, made no difference to the deprivation effect (table E5).

Adjusting for B. cenocepacia status

3.5% (156/4445) of individuals in the %FEV1 analysis for the <18 group were recorded as having B. cenocepacia. Addition of this variable to the model for %FEV1 made no difference to the deprivation effect (table E5).

Table E5: Additional models fitted as robustness tests in response to reviewers comments, based upon the final FEV1 model in appendix E2.

	final	IMD as time varying	Final + care center	Data <2000 excluded	Cepacia added
Constant	95.688***	95.726***	89.83***	95.609***	95.641***
	(1.276)	(1.274)	(1.88)	(1.303)	(1.274)
Age-5	-1.719***	-1.719***	-1.69***	-1.722***	-1.705***
	(0.085)	(0.085)	(0.09)	(0.089)	(0.085)
Number of F508 alleles: 0/2	-0.142	-0.135	-0.54	-0.526	-0.140
	(1.402)	(1.402)	(1.38)	(1.452)	(1.401)
Number of F508 alleles: 1/2	-0.258	-0.260	-0.19	-0.258	-0.237
	(0.829)	(0.829)	(0.81)	(0.863)	(0.828)
Male	-0.924	-0.921	-1.11	-1.073	-0.919
	(0.768)	(0.768)	(0.75)	(0.800)	(0.767)
nonwhite	-7.256***	-7.248***	-7.36***	-6.635**	-7.176***
	(2.149)	(2.148)	(2.13)	(2.203)	(2.146)
Screened	-3.599***	-3.581***	-2.63*	-3.809***	-3.601***
	(1.064)	(1.064)	(1.15)	(1.101)	(1.062)
Deprivation score	-0.071***		-0.07***	-0.075***	-0.071***
	(0.016)		(0.02)	(0.016)	(0.016)
Age-5 x nallele: 0/2	0.016	0.017	0.09	0.065	0.014
	(0.161)	(0.161)	(0.16)	(0.166)	(0.160)
Age-5x nallele: 1/2	0.242*	0.242*	0.25*	0.228*	0.242*
	(0.100)	(0.100)	(0.10)	(0.104)	(0.100)
Age-5 x sex: Male/Female	0.293**	0.292**	0.29**	0.302**	0.292**
	(0.094)	(0.094)	(0.09)	(0.098)	(0.094)
Age-5 x nonwhite	0.353	0.354	0.31	0.285	0.343
	(0.266)	(0.266)	(0.27)	(0.273)	(0.266)
Age-5 x screened	0.634***	0.631***	0.53***	0.667***	0.637***
	(0.131)	(0.131)	(0.13)	(0.136)	(0.131)
Deprivation score (time varying)		-0.073***			
		(0.016)			
Cepacia					-2.302
					(2.415)
ageminusfive x Cepacia					-0.194
					(0.271)
Log-likelihood	-80509.181	-80508.314	-80316.11	-74959.478	-80499.082
Deviance	161018.362	161016.629	160632.21	149918.955	160998.164
AIC	161100.362	161098.629	160976.21	149994.955	161084.164
BIC	161424.362	161422.628	162335.43	150292.483	161423.969
N	19979	19979	19979	18577	19979
Groups	4445	4445	4445	4334	4445

Appendix references

1. Adler AI, Shine BS, Chamnan P, Haworth CS, Bilton D. Genetic determinants and epidemiology of cystic fibrosis-related diabetes: results from a British cohort of children and adults. Diabetes Care. 2008; **31**(9): 1789-94.
2. Chamnan P, Shine BS, Haworth CS, Bilton D, Adler AI. Diabetes as a determinant of mortality in cystic fibrosis. Diabetes Care. 2010; **33**(2): 311-6.
3. Mehta G, Sims EJ, Culross F, McCormick JD, Mehta A. Potential benefits of the UK Cystic Fibrosis Database. J R Soc Med. 2004; **97 Suppl 44**: 60-71.
4. CF Trust. CF Registry - Annual Data Reports. http://www.cftrust.org.uk/aboutcf/publications/cfregistryreports/ (accessed 2nd December 2012). 2012.
5. Cystic Fibrosis Trust. UK CF Registry annual data report 2010. http://www.cftrust.org.uk/aboutcf/publications/cfregistryreports/ (accessed 28th September 2012). 2011.
6. Taylor-Robinson D, Whitehead M, Diderichsen F, Olesen HV, Pressler T, Smyth RL, et al. Understanding the natural progression in %FEV1 decline in patients with cystic fibrosis: a longitudinal study. Thorax. 2012; **67**(10): 860-6.
7. Miller MR, Hankinson J, Brusasco V, Burgos F, Casaburi R, Coates A, et al. Standardisation of spirometry. Eur Respir J. 2005; **26**(2): 319-38.
8. Wang X, Dockery DW, Wypij D, Fay ME, Ferris BG, Jr. Pulmonary function between 6 and 18 years of age. Pediatr Pulmonol. 1993; **15**(2): 75-88.
9. Hankinson JL, Odencrantz JR, Fedan KB. Spirometric reference values from a sample of the general U.S. population. Am J Respir Crit Care Med. 1999; **159**(1): 179-87.
10. Taylor-Robinson D, Agarwal U, Diggle PJ, Platt MJ, Yoxall B, Alfirevic Z. Quantifying the impact of deprivation on preterm births: a retrospective cohort study. PLoS One. 2011; **6**(8): e23163.
11. Semple MG, Taylor-Robinson DC, Lane S, Smyth RL. Household tobacco smoke and admission weight predict severe bronchiolitis in infants independent of deprivation: prospective cohort study. PLoS One. 2011; **6**(7): e22425.
12. Bergen H, Hawton K, Waters K, Ness J, Cooper J, Steeg S, et al. Premature death after self-harm: a multicentre cohort study. Lancet. 2012; **380**(9853): 1568-74.
13. DH. Improving outcomes and supporting transparency. Part 1: A public health outcomes framework for England, 2013-2016 http://www.dh.gov.uk/prod_consum_dh/groups/dh_digitalassets/@dh/@en/documents/digitalasset/dh_132559.pdf (accessed 3 December 2012). 2012.
14. ONS. Indices of Deprivation across the UK http://www.neighbourhood.statistics.gov.uk/dissemination/Info.do?page=analysisandguidance/analysisarticles/indices-of-deprivation.htm (accessed 29th July 2011). 2011.
15. Krieger N, Waterman P, Chen JT, Soobader MJ, Subramanian SV, Carson R. Zip code caveat: bias due to spatiotemporal mismatches between zip codes and US census-defined geographic areas--the Public Health Disparities Geocoding Project. Am J Public Health. 2002; **92**(7): 1100-2.
16. Diggle P, Heagerty P, Liang K-Y, Zeger SL. Analysis of Longitudinal Data (second edition). Oxford: Oxford University Press; 2002.
